# Radical reactivity of antiaromatic Ni(II) norcorroles with azo radical initiators

**DOI:** 10.3762/bjoc.20.172

**Published:** 2024-08-12

**Authors:** Siham Asyiqin Shafie, Ryo Nozawa, Hideaki Takano, Hiroshi Shinokubo

**Affiliations:** 1 Department of Molecular and Macromolecular Chemistry, Graduate School of Engineering and Integrated Research Consortium Chemical Sciences (IRCCS), Nagoya University, Furo-cho, Chikusa-ku, Nagoya, Aichi 464-8603, Japan; 2 Institute for Advanced Research, Nagoya University, Furo-cho, Chikusa-ku, Nagoya, Aichi 464-8601, Japan

**Keywords:** 16π, antiaromatic, norcorrole, porphyrinoid, radical

## Abstract

Norcorrole is a stable 16π-antiaromatic porphyrinoid that exhibits characteristic reactivities and physical properties. Here, we disclose the reaction of Ni(II) norcorroles with alkyl radicals derived from azo radical initiators. The radical selectively attacked the distal α-position relative to the *meso*-position to construct a nonaromatic bowl-shaped structure. The photophysical and electrochemical properties of the obtained radical adducts were compared to those of the parent Ni(II) norcorrole. The radical reactivity of Ni(II) norcorroles was investigated by density functional theory (DFT) calculations.

## Introduction

**Figure 1:**
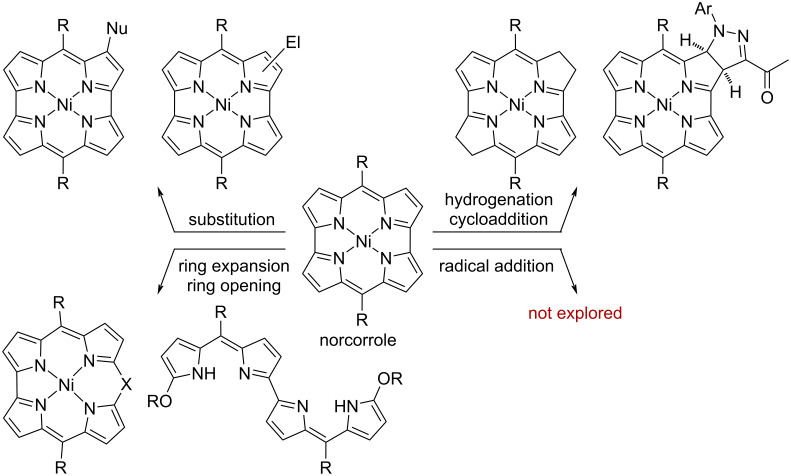
Reactivities of norcorroles with various reagents.

Considerable attention has been directed toward antiaromatic norcorroles [1–3] due to the fascinating physical properties, such as reversible redox properties [4–5] and stacked-ring aromaticity [6–10]. While Ni(II) norcorroles are stable under ambient conditions despite the distinct 16π-antiaromaticity, they show unique reactivities with various reagents due to the high-lying HOMO and low-lying LUMO (Figure 1) [11]. Reactions with nucleophiles (Nu) proceed with perfect regioselectivity at the distal β-position relative to the *meso*-position [12–15]. On the other hand, reactions with electrophiles (El) also occur preferentially at the β-positions, but the regioselectivity depends on the electrophile [16–18]. In addition, C–C double bonds of the norcorrole skeleton outside the π-delocalization pathway exhibit a reactivity similar to an alkene to afford hydrogenated norcorroles by hydrogenation [19] or reduction with hydrazine [20] and [3 + 2]-cycloadducts with 1,3-dipoles [21]. Moreover, the ring-expansion or ring-opening reactions of Ni(II) norcorroles are induced by an activated zwitterionic intermediate [22], oxidants [23–24], and carbenes [25–26].

During the last decade, the various reactivities of Ni(II) norcorroles have been elucidated. However, the reaction with radical species has remained unexplored. Here, we disclose the radical functionalization of Ni(II) norcorroles with simple and frequently used azo radical initiators to furnish nonconjugated macrocycles with bowl-shaped structures [27]. The photophysical and electronic properties of the obtained products are also presented. We also discuss the selectivity of the radical addition to Ni(II) norcorroles using DFT calculations.

## Results and Discussion

### Reactivity with azo radical initiators

**Scheme 1:**
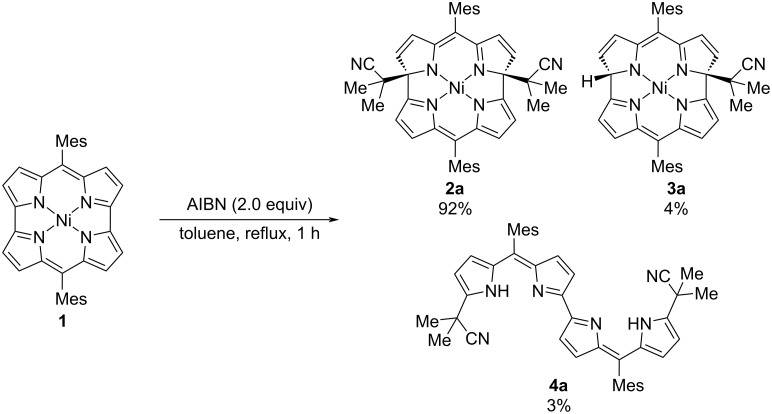
Reaction of norcorrole **1** with AIBN.

We selected 2,2'-azobis(isobutyronitrile) (AIBN) as a radical source. Ni(II) dimesitylnorcorrole **1** was treated with AIBN in refluxing toluene (Scheme 1). The reaction smoothly proceeded to afford dialkylated macrocycle **2a** in 92% yield. In addition to **2a**, monoalkylated product **3a** and dipyrrin dimer **4a** were obtained as minor products in 4% and 3% yield, respectively.

**Figure 2:**
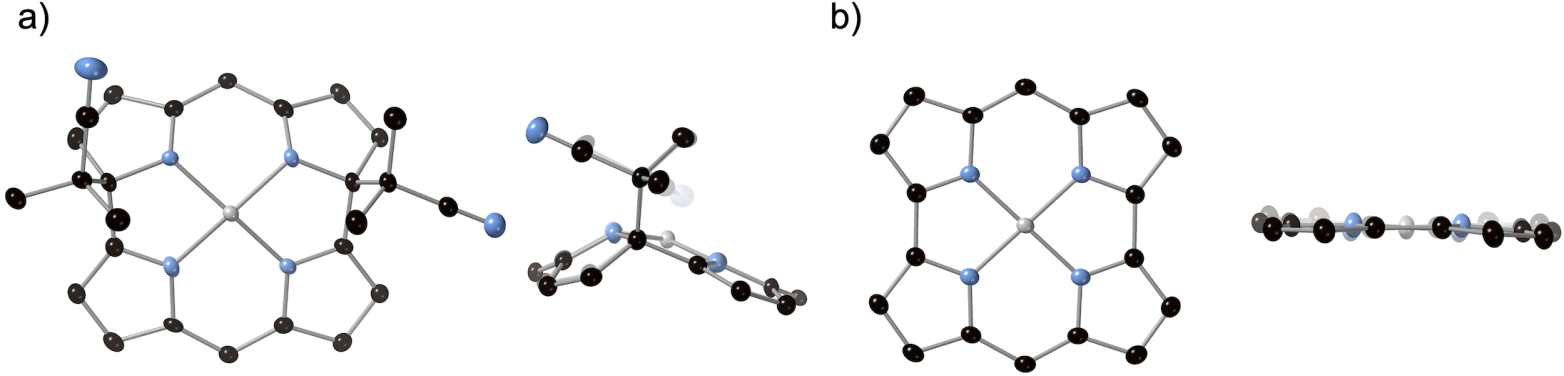
Top and side views of the X-ray structures of a) **2a** and b) **1** [2]. Mesityl groups and hydrogen atoms were omitted for clarity. Thermal ellipsoids are drawn at 50% probability.

The structure of **2a** was unambiguously confirmed by single-crystal X-ray analysis, which revealed that two alkyl substituents were located on the same side of the molecule (Figure 2a). Compared to the planar structure of **1** (Figure 2b) [2], **2a** displays a nonplanar structure due to the sp^3^ carbon atoms adjacent to the nitrogen atoms. The ^1^H NMR spectrum of **2a** confirmed that the antiaromatic character of the macrocycle changed to nonaromatic upon radical addition (see Supporting Information File 1).

**Scheme 2:**
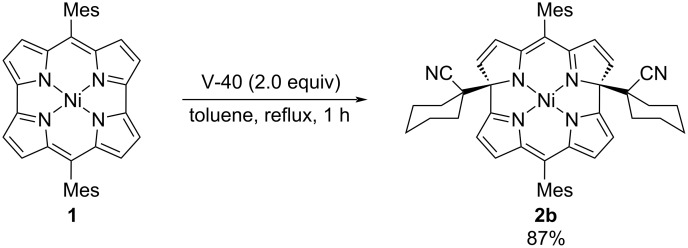
Reaction of norcorrole **1** with V-40.

1,1'-Azobis(cyclohexane-1-carbonitrile) (V-40) was also examined as a radical source. The reaction afforded **2b** in 87% yield (Scheme 2). Unfortunately, other radical sources, such as benzoyl peroxide, TEMPO, and the combination of alkyl halides with BEt_3_, were not applicable to this reaction.

### Physical properties

**Figure 3:**
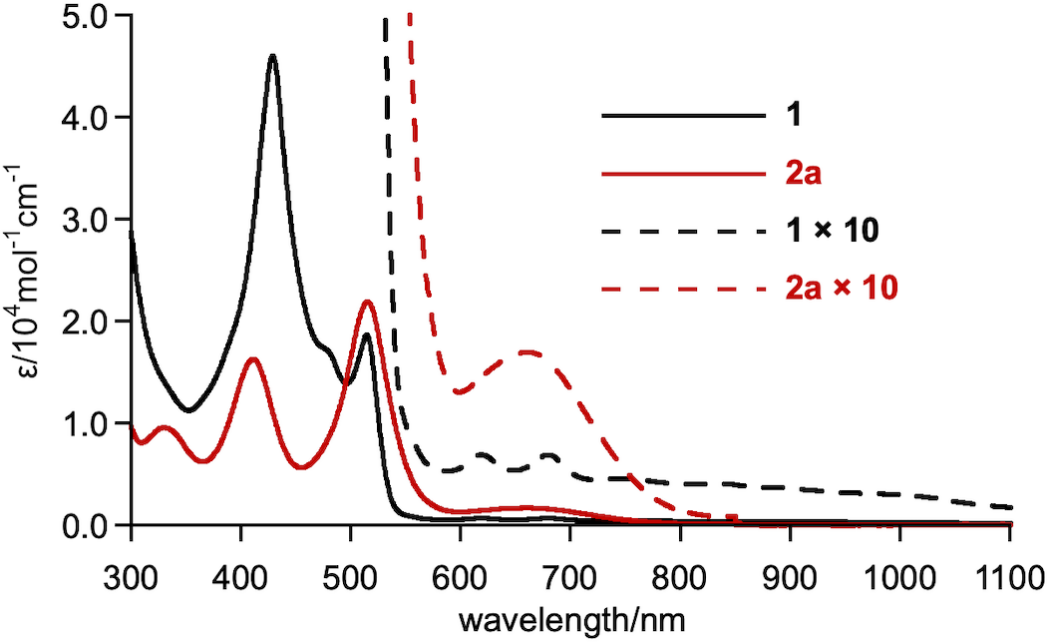
UV–vis–NIR absorption spectra of **1** and **2a** in CH_2_Cl_2_.

The electronic absorption spectra of norcorrole **1** and adduct **2a** are shown in Figure 3. While norcorrole **1** exhibited a weak absorption band from 600 nm to the NIR region, due to the characteristic forbidden HOMO–LUMO transition of the antiaromatic compound, nonconjugated macrocycle **2a** did not possess such an absorption band, indicating the loss of antiaromaticity in **2a**. Macrocycle **2a** possessed new absorption bands from 600 to 800 nm. The simulated absorption spectrum of **2a** obtained by TD DFT calculations at the M06/6-31G(d)+SDD//B3LYP-D3/6-31G(d)+SDD level of theory was consistent with the experimental results. Therein, the absorption band at 670 nm (*f* = 0.0026) was attributed to the transition from HOMO to LUMO+1.

**Figure 4:**
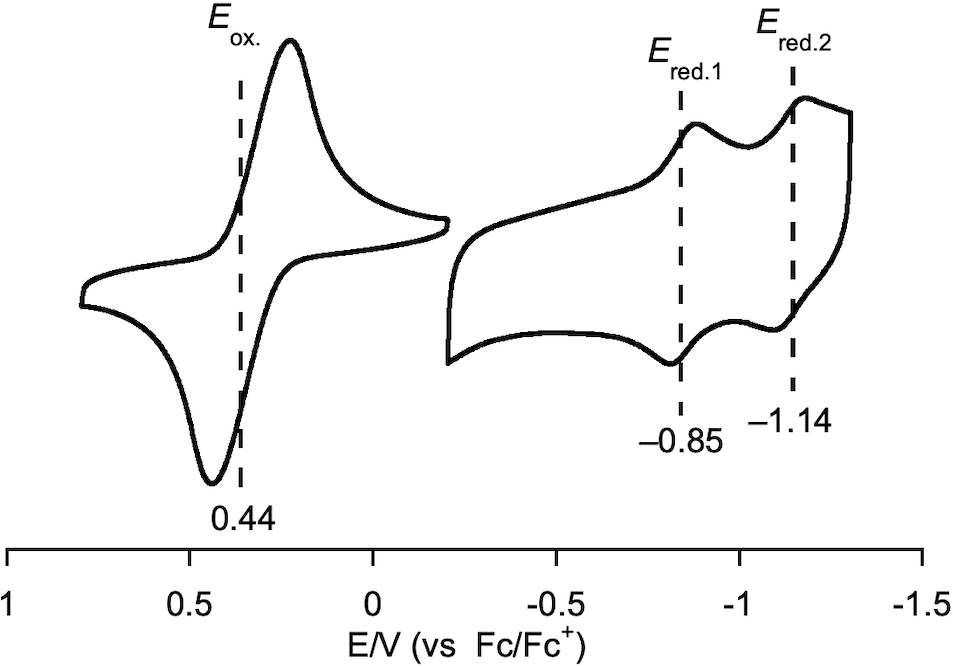
Cyclic voltammogram of **2a** in CH_2_Cl_2_. Supporting electrolyte: 0.1 M Bu_4_NPF_6_; working electrode: glassy carbon; counter electrode: Pt; reference electrode: Ag/AgNO_3_; scan rate: 50 mV⋅s^−1^.

Next, the electrochemical properties of **2a** in CH_2_Cl_2_ were examined using cyclic voltammetry (Figure 4). Macrocycle **2a** exhibited one reversible oxidation wave at 0.44 V and two reversible reduction waves at −0.85 V and −1.14 V. The electrochemical HOMO–LUMO gap of **2a** is 1.29 V, which is larger than that of **1a** (1.08 V) [2].

### DFT calculations

**Scheme 3:**
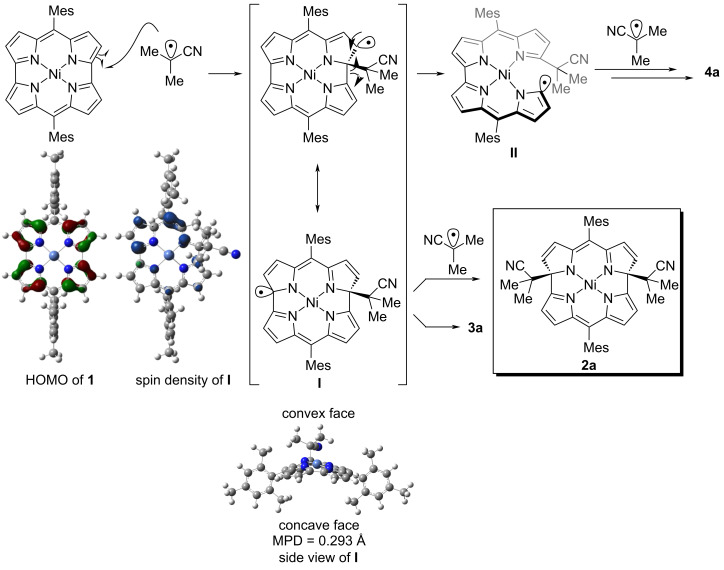
Plausible reaction mechanism.

We next conducted DFT calculations using Gaussian 16 [28] to elucidate the reactivity of Ni(II) norcorroles with radical species (Scheme 3). All calculations for the ground state were performed at the (U)B3LYP-D3/6-31G(d)+SDD level of theory. The SOMO of an isobutyronitrile radical (−5.98 eV), which was generated through denitrogenation of AIBN, is closer to the HOMO level of Ni(II) norcorrole **1** (−4.68 eV) rather than its LUMO (−3.16 eV). This result explains the selective addition of the electrophilic isobutyronitrile radical to the distal α-position of the pyrrole unit. The calculated molecular orbital coefficient of the HOMO indicates that two α-carbon atoms of the pyrrole subunits are the most reactive positions for electrophilic species. In addition, the distal α-carbon atom relative to the *meso*-position could be more reactive than the proximal α-carbon atom due to the steric hindrance of bulky mesityl groups. Consequently, the isobutyronitrile radical predominantly attacks the distal α-carbon atom relative to the *meso*-position to afford the corresponding radical intermediate **I**. The calculated spin density of radical **I** revealed a substantial radical character at the α-position of the pyrrole skeleton. Finally, another isobutyronitrile radical reacts with **I** at the convex face to form the major product **2a,** with two alkyl substituents on the same side of the molecule. The mean-plane deviation (MPD) of **I** was 0.293 Å, where the mean plane was defined by carbon, nitrogen, and nickel atoms of the norcorrole core. For the byproducts, **3a** would be generated through the quenching of radical **I** with a hydrogen atom source. Bisdipyrrin **4a** could be formed through the ring-opening reaction of **I** by the homolytic cleavage of the C(sp^2^)–C(sp^2^) bond to radical **II**, the addition of the isobutyronitrile radical, and subsequent demetallation.

## Conclusion

In conclusion, we have investigated the addition reaction of electrophilic alkyl radicals derived from azo radical initiators to antiaromatic Ni(II) norcorroles. The reaction smoothly proceeded to afford bowl-shaped nonconjugated macrocycles **2a** in excellent yield, which exhibited markedly different photophysical and electrochemical properties with norcorrole **1**. The intrinsic reactivities of Ni(II) norcorroles with neutral radical species were revealed by DFT calculations, where populations of the HOMO of the norcorrole unit and the spin density of the radical intermediate governed the regioselectivity.

## Supporting Information

File 1 Experimental procedures, compound characterization data including NMR and MS spectra, additional crystal data and details from DFT calculations.

## Data Availability

All data that supports the findings of this study is available in the published article and/or the supporting information to this article.
