# Supplementary material for: Radical reactivity of antiaromatic Ni(II) norcorroles with azo radical initiators
Source: Beilstein J Org Chem. 2024 Aug 12;20:1967–72. doi: 10.3762/bjoc.20.172 (PMC11331536; doi:10.3762/bjoc.20.172)
Supplement: File 1 — Experimental procedures, compound characterization data including NMR and MS spectra, additional crystal data and details from DFT calculations. [file Beilstein_J_Org_Chem-20-1967-s001.pdf]

BEILSTEIN JOURNAL OF ORGANIC CHEMISTRY

Supporting Information

for

Radical reactivity of antiaromatic Ni(II) norcorroles with azo radical initiators

Siham Asyiqin Shafie, Ryo Nozawa, Hideaki Takano and Hiroshi Shinokubo

Beilstein J. Org. Chem. **2024,** *20,* 1967–1972. doi:10.3762/bjoc.20.172

Experimental procedures, compound characterization data including NMR and MS spectra, additional crystal data and details from DFT calculations

License and Terms: This is a supporting information file under the terms of the Creative Commons Attribution License (https://creativecommons.org/licenses/by/4.0). Please note that the reuse, redistribution and reproduction in particular requires that the author(s) and source are credited and that individual graphics may be subject to special legal provisions.
The license is subject to the *Beilstein Journal of Organic Chemistry* terms and conditions: (https://www.beilstein-journals.org/bjoc/terms)

Table of Contents

1. Instrumentation and materials S2
2. Experimental procedures and compound data S3
3. NMR spectra S5
4. Mass spectra S9
5. Crystal data S11
6. DFT Calculations S12
7. References S22

1. Instrumentation and materials

^1H NMR (500 MHz) and ^{13}C NMR (126 MHz) spectra were recorded on a Bruker AVANCE III HD spectrometer. Chemical shifts were reported as the delta scale in ppm relative to $CHCl_3$ (δ = 7.26 ppm) for ^1H NMR and $CDCl_3$ (δ = 77.16 ppm) for ^{13}C NMR.

UV–vis–NIR absorption spectra were recorded on a Shimadzu UV-2550 or JASCO V 670 spectrometer.

High-resolution and electron spray ionization time-of-flight (ESI-TOF) mass spectra were taken on a Bruker micrOTOF instrument using a positive ionization mode.

X-ray data were obtained using a Bruker D8 QUEST X-ray diffractometer with an IμS microfocus X-ray source and a PHOTON II detector.

Cyclic voltammograms were obtained under the following conditions: solvent: CH_2Cl_2, electrolyte: 0.1 M Bu_4NPF_6, working electrode: glassy carbon, counter electrode: Pt, reference electrode: $Ag/AgNO_3$, scan rate: 50 mV s^{-1}.

Dry toluene was purchased from KANTO CHEMICAL CO., INC. as a dehydrated grade. AIBN and V-40 were purchased from Wako Pure Chemical Industries, Ltd. Ni(II) mesitylnorcorrole **1** was prepared according to the literature.[1]

Unless otherwise noted, materials obtained from commercial suppliers were used without further purification.

2. Experimental procedures and compound data

Synthesis of dialkylated macrocycle 2a

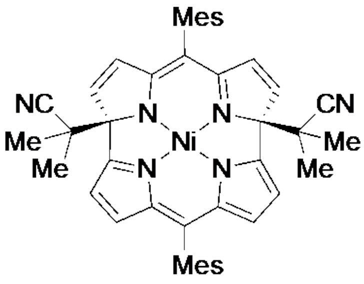

2a

To a Schlenk flask was added compound **1** (23.0 mg, 40 μmol), AIBN (13.2 mg, 80 μmol) and dry toluene (4 mL) under N_2 atmosphere. The reaction mixture was stirred at reflux for 1 h. After removing the solvent in vacuo, the mixture was purified by PTLC (eluent: CH_2Cl_2/hexane = 1:2) and compound **2a** (26.2 mg, 37 μmol, 92%) was obtained as a maroon solid.

^{1}H NMR (500 MHz, $CDCl_3$, 298 K): δ = 7.19 (d, J = 5.4 Hz, 2H), 6.93 (s, 1H), 6.92 (s, 1H), 6.91 (s, 1H), 6.87 (s, 1H), 6.49 (d, J = 4.2 Hz, 2H), 6.46 (d, J = 4.2 Hz, 2H), 6.09 (d, J = 5.4 Hz, 2H), 2.34 (s, 3H), 2.31 (s, 3H), 2.19 (s, 3H), 2.18 (s, 3H), 2.14 (s, 3H), 1.86 (s, 3H), 1.60 (s, 6H), 1.42 (s, 6H) ppm; ^{13}C NMR (126 MHz, $CDCl_3$, 298 K): δ = 174.3, 153.4, 145.8, 145.6, 140.7, 139.0, 137.9, 136.9,136.4, 136.3, 133.5, 133.3, 132.7, 131.9, 128.3, 128.2, 128.0, 127.9, 127.7, 123.7, 115.2, 98.0, 92.4, 40.1, 24.4, 23.0, 21.3, 21.2, 21.2, 21.2, 20.3, 20.0, ppm; HRMS (ESI): $[M+Na]^+$ Calcd for $(C_{44}H_{42}N_6Ni)Na^+$: 735.2717; Found: 735.2721

Synthesis of dialkylated macrocycle 2b

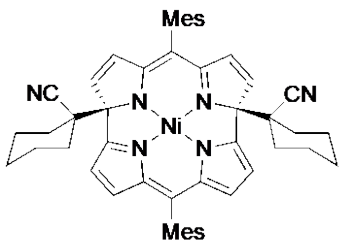

2b

To a Schlenk flask was added compound **1** (23.0 mg, 40 µmol), V–40 (19.5 mg, 80 µmol) and dry toluene (4 mL) under N_2 atmosphere. The reaction mixture was stirred at reflux for 1 h. After removing the solvent in vacuo, the mixture was purified by PTLC (eluent: CH_2Cl_2/hexane = 1:1) and compound **2b** (27.4 mg, 35 µmol, 87%) was obtained as a maroon solid.

^{1}H NMR (500 MHz, $CDCl_3$, 298 K): δ = 7.18 (d, J = 5.4 Hz, 2H), 6.93–6.90 (m, 3H), 6.86 (s, 1H), 6.49 (d, J = 4.3 Hz, 2H), 6.44 (d, J = 4.3 Hz, 2H), 6.06 (d, J = 5.4 Hz, 2H), 2.33 (s, 3H), 2.30 (s, 3H), 2.24–2.15 (m, 13H), 1.94–1.86 (m, 2H) , 1.85–1.78 (m, 2H), 1.82 (s, 3H), 1.78–1.69 (m, 4H), 1.69–1.58 (m, 4H), 1.32–1.22 (m, 4H) ppm; ^{13}C NMR (126 MHz, $CDCl_3$, 298 K): δ = 174.7, 153.6, 145.8, 145.3, 140.7, 139.1, 137.8, 136.8, 136.5, 136.4, 133.7, 133.4, 132.6, 132.0, 128.2, 128.2, 128.0, 127.9, 127.7, 122.1, 115.3, 98.2, 92.9, 47.4, 32.4, 30.6, 25.0, 23.7, 23.4, 21.5, 21.3, 21.2, 21.1, 20.5, 19.9 ppm; HRMS (ESI): $[M+Na]^+$ Calcd for $(C_{50}H_{50}N_6Ni)Na^+$: 815.3343; Found: 815.3348

3. NMR spectra

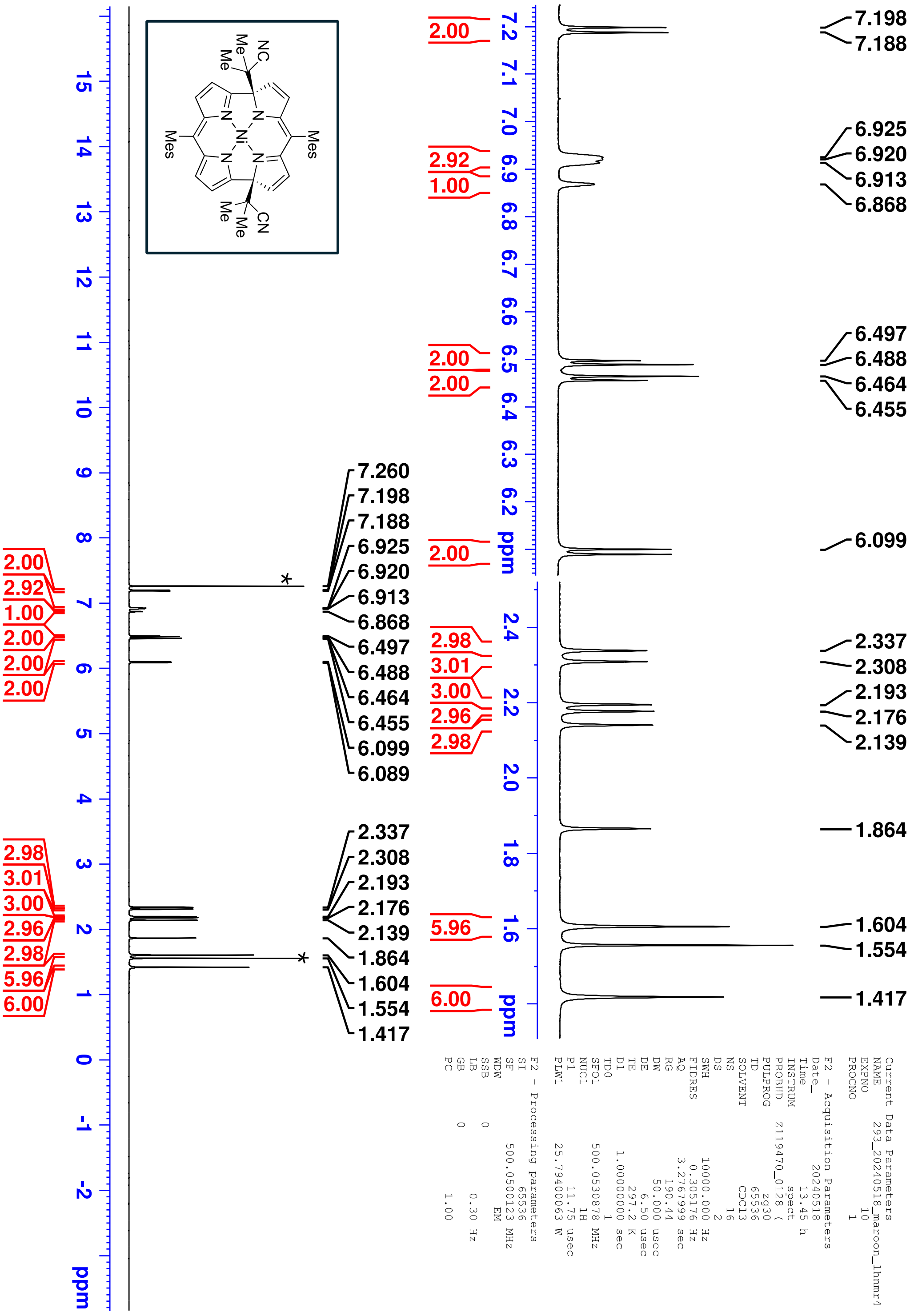

Figure S1. ^{1}H NMR spectrum of **2a** in $CDCl_3$ at 25 °C.

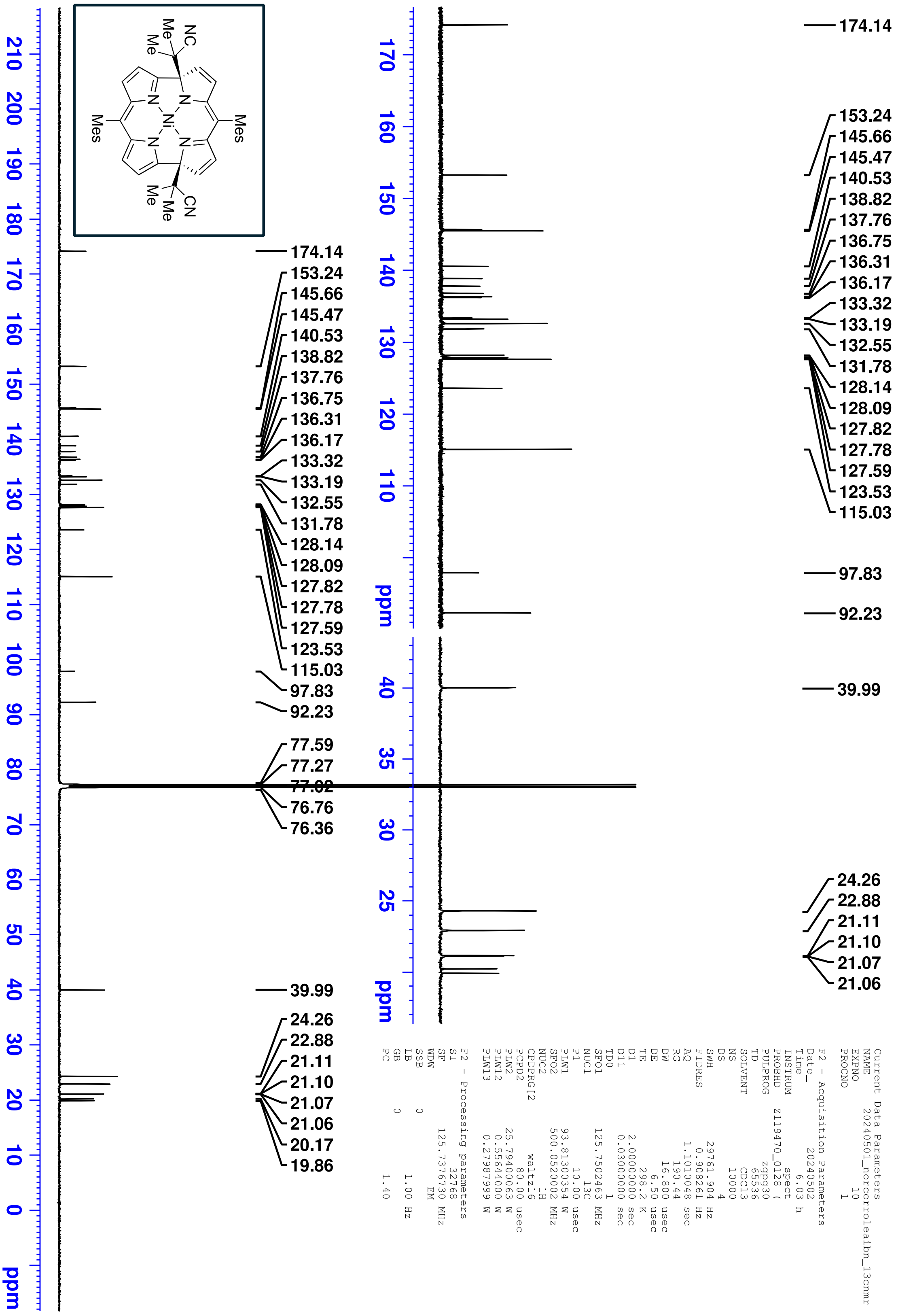

Figure S2. ^{13}C NMR spectrum of **2a** in $CDCl_3$ at 25 °C.

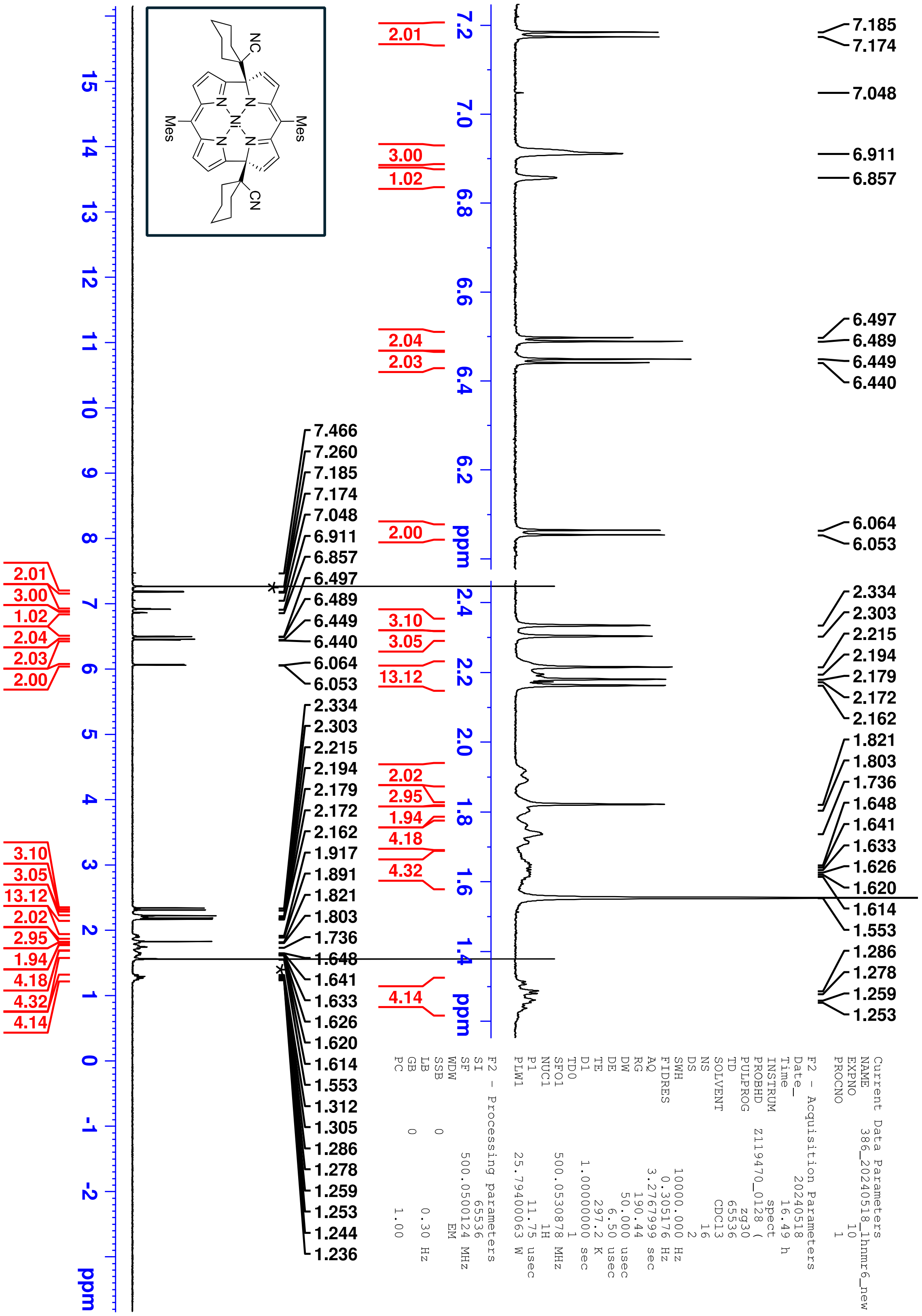

Figure S3. ^{1}H NMR spectrum of **2b** in $CDCl_3$ at 25 °C.

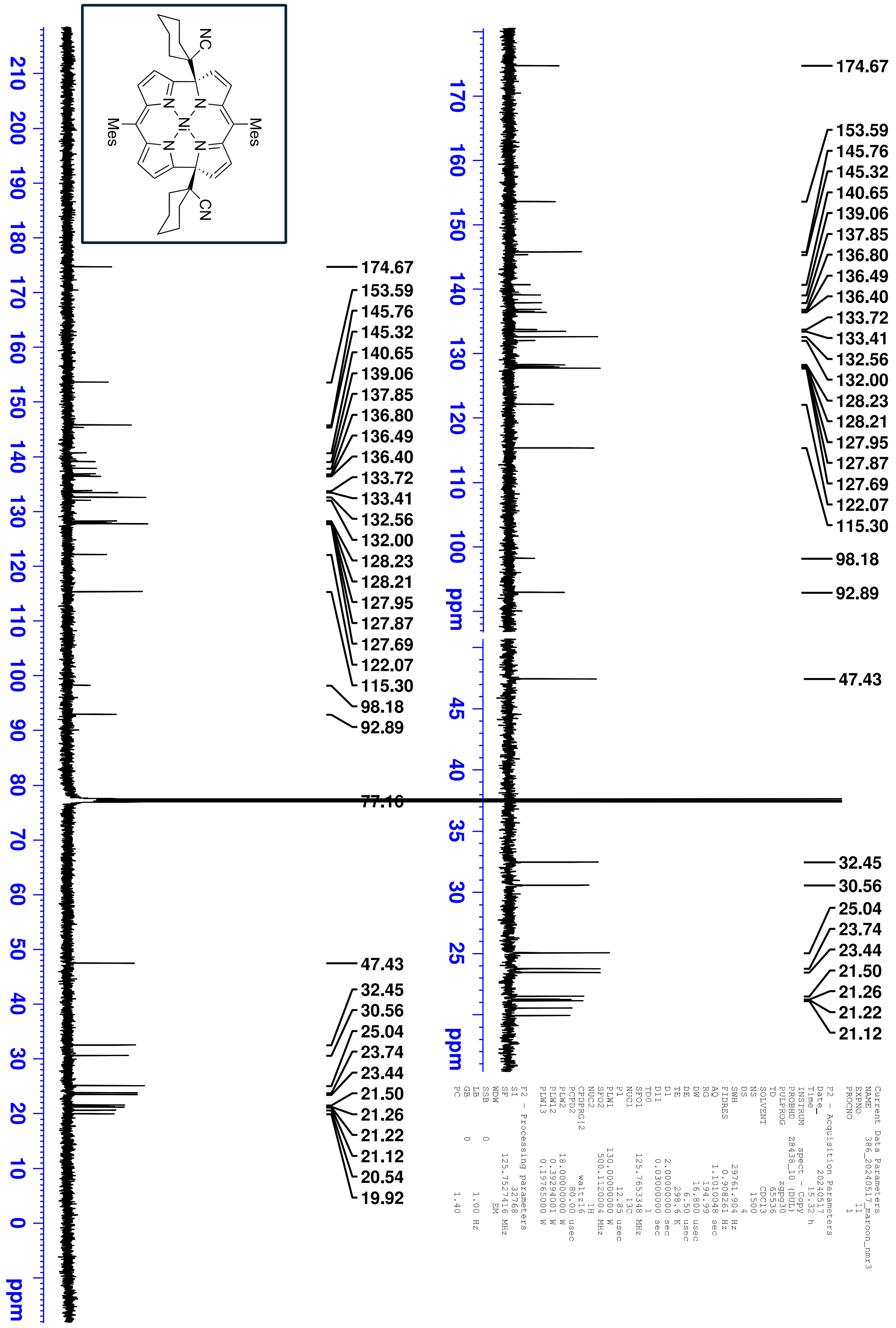

Figure S4. ^{13}C NMR spectrum of **2b** in $CDCl_3$ at 25 °C.

4. Mass spectra

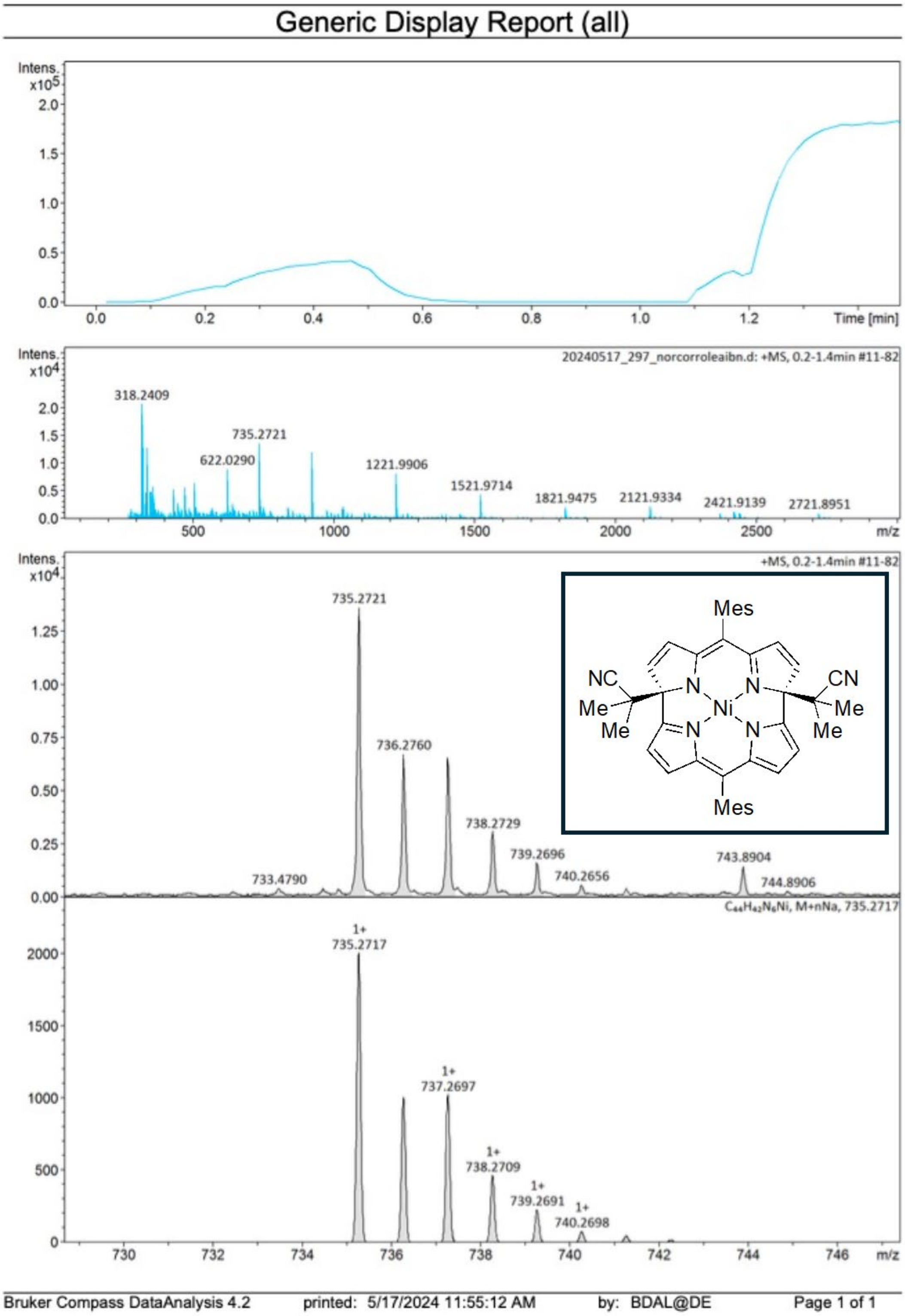

Figure S5. ESI-TOF mass spectrum of **2a**.

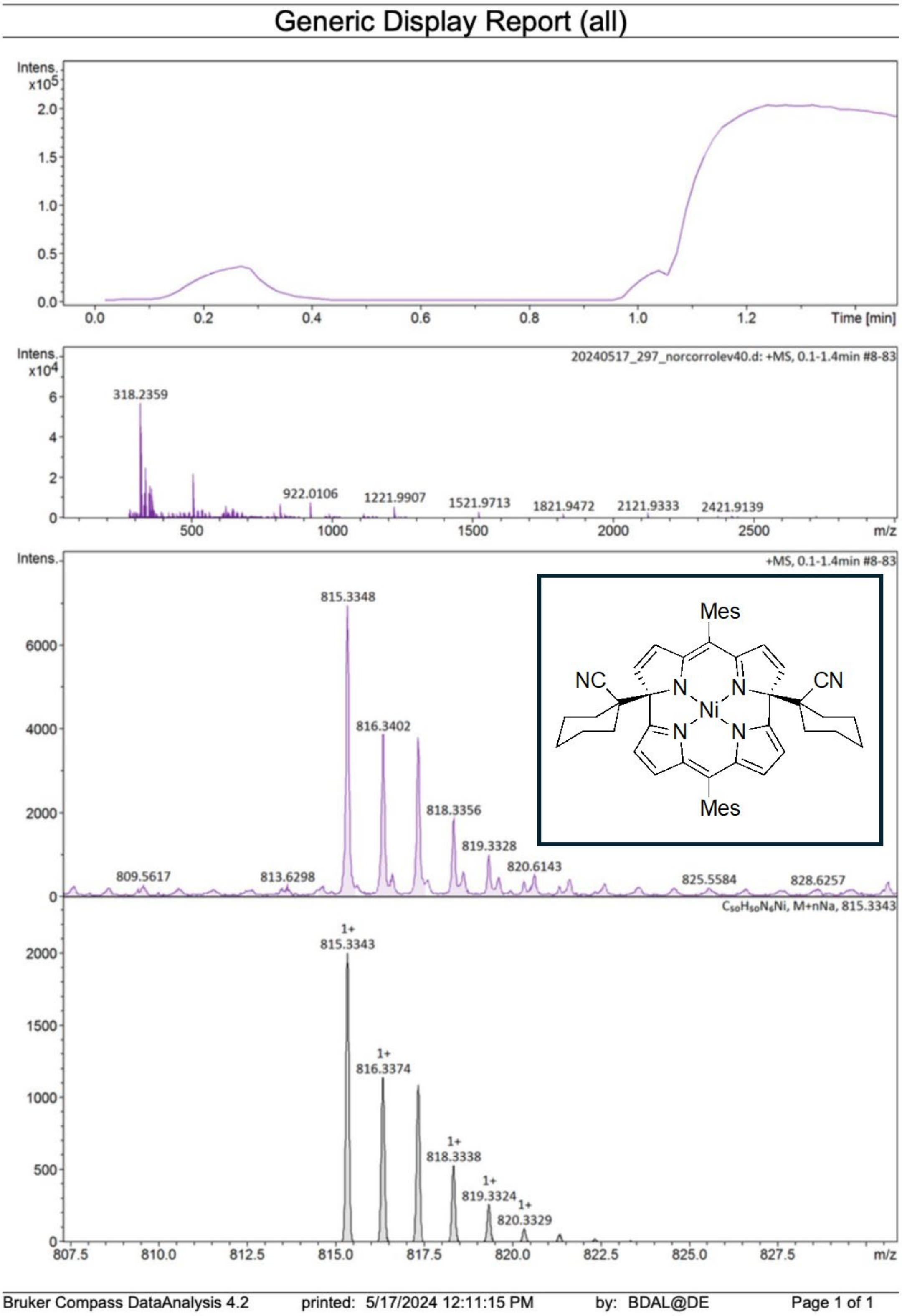

Figure S6. ESI-TOF mass spectrum of **2b**.

5. Crystal data

Crystallographic data for 2a: Single crystals were obtained by vapour diffusion of acetonitrile into a chloroform solution of **2a**. $C_{44}H_{42}N_6Ni$, M_w = 713.54, monoclinic, space group $P2_1/c$, a = 23.594(5), b = 15.092(5), c = 10.888(5) Å, $\alpha = \gamma$ = 90.000(5)°, β = 101.917(4)°, V = 3793(2) Å^3, Z = 4, D_{calc} = 1.249 g/cm^3, T = 93 K, R_1 = 0.0433 (I > 2.0 $\sigma(I)$), wR_2 = 0.0945 (all data), GOF = 1.037, CCDC No. = 2356750.

Table S1. Crystallographic data of **2a**

compound	**2a**
Formula	$C_{44}H_{42}N_6Ni$
Formula weight	713.54
Crystal system	monoclinic
Space group	$P2_1/c$ (No. 14)
Crystal color	green
Crystal description	block
a [Å]	23.594(5)
b [Å]	15.092(5)
c [Å]	10.888(5)
α [°]	90.000(5)
β [°]	101.917(4)
γ [°]	90.000(5)
V [Å^3]	3793.(2)
Z	4
d_{calcd} [g cm^{-3}]	1.249
R_1 ($I > 2\sigma(I)$)	0.0433
wR_2 (all data)	0.0945
Goodness-of-fit	1.037
Temperature [K]	93(2)
Solvent	$CHCl_3$/MeCN
CCDC No.	2356750

6. DFT Calculations

Cartesian coordinate of the optimized structures

Isobutyronitrile radical

C	-1.32841100	3.08648100	0.26478700
C	0.15290600	2.95254200	0.46721800
H	-1.72947300	3.99366900	0.72299500
H	-1.56999700	3.10297900	-0.80803400
H	-1.85469400	2.21993900	0.69106400
C	0.86450200	1.75908900	-0.10069700
H	1.93605500	1.77480700	0.11219100
H	0.44256000	0.82932800	0.30806900
H	0.72743000	1.71247200	-1.19093400
C	0.85769700	3.92266800	1.17260600
N	1.45376700	4.74315100	1.76919200

Norcorrole (**1**)

C	0.74868700	2.56943500	0.49147600
C	1.86096300	3.41251400	0.18722600
C	2.99609000	2.59730200	0.17826000
C	2.57314500	1.25511100	0.46876400
N	1.22006700	1.32519300	0.68673700
H	1.82830700	4.47491100	-0.01432000
H	4.01308600	2.90312500	-0.02937600
C	-0.74597800	2.57016500	0.49177000
C	-1.85754600	3.41432500	0.18794000
N	-1.21849600	1.32638400	0.68722700
C	-2.99347200	2.60021900	0.17941900
H	-1.82393300	4.47668600	-0.01363300
C	-2.57172500	1.25762100	0.46977900
H	-4.01024800	2.90702800	-0.02783300
C	2.57201500	-1.25790800	0.46962700
C	2.99375500	-2.60091900	0.18117700
C	1.85779700	-3.41496100	0.19039800

C	0.74618300	-2.57036800	0.49294500
N	1.21870700	-1.32637000	0.68673700
H	4.01058100	-2.90808500	-0.02525300
H	1.82420400	-4.47760000	-0.00971100
C	-0.74849300	-2.56963700	0.49324900
C	-1.86105800	-3.41315100	0.19117700
N	-1.21972300	-1.32517800	0.68721800
C	-2.99622400	-2.59800300	0.18242100
H	-1.82858400	-4.47582600	-0.00892900
C	-2.57305500	-1.25539800	0.47067100
H	-4.01343600	-2.90418200	-0.02357700
C	3.23732100	-0.00174300	0.37771400
C	-3.23716800	0.00141400	0.37901200
Ni	0.00016000	0.00002800	0.79164500
C	4.69905900	-0.00250900	0.07036300
C	5.13292900	-0.00776900	-1.26948200
C	5.62815600	0.00773200	1.12824700
C	6.50708300	-0.00415700	-1.52825000
C	6.99239700	0.01085700	0.82296200
C	7.45202000	0.00193200	-0.49762500
H	6.84678700	-0.00473600	-2.56212300
H	7.71332800	0.02179100	1.63811000
C	-4.69902800	0.00208000	0.07224800
C	-5.62769000	0.01361500	1.13050100
C	-5.13344200	-0.00312700	-1.26742100
C	-6.99204900	0.01806600	0.82576200
C	-6.50769600	0.00184300	-1.52564000
C	-7.45221200	0.00922400	-0.49463800
H	-7.71264100	0.02999100	1.64119600
H	-6.84781600	0.00132000	-2.55937600
C	-5.15427500	0.02499200	2.56593400
H	-4.53459100	-0.85287300	2.78462500
H	-4.53541500	0.90660700	2.77137000

H	-5.99814400	0.02975900	3.26238400
C	-4.13397700	-0.00882400	-2.40155600
H	-3.47919900	0.86942100	-2.35540400
H	-3.48470200	-0.89087100	-2.35108600
H	-4.63755800	-0.00967000	-3.37294000
C	-8.93206800	-0.02063400	-0.79903900
H	-9.30127200	-1.05314300	-0.86156000
H	-9.51265200	0.48508900	-0.01981200
H	-9.15508600	0.46263700	-1.75647500
C	4.13300300	-0.01210300	-2.40321600
H	3.48267900	-0.89336800	-2.35260200
H	3.47931000	0.86693000	-2.35668400
H	4.63619300	-0.01343100	-3.37480200
C	5.15532800	0.01917800	2.56387300
H	4.53739300	0.90133000	2.76979200
H	4.53489200	-0.85815200	2.78257900
H	5.99948000	0.02295400	3.25998500
C	8.93172100	-0.02937900	-0.80263400
H	9.29980000	-1.06225400	-0.86572200
H	9.15486700	0.45404000	-1.75996600
H	9.51315600	0.47541200	-0.02343900

Intermediate **I**

C	0.77559600	2.53266100	-1.23699100
C	1.81163500	3.19846700	-0.35442000
C	2.82628300	2.33689900	-0.13757000
C	2.46827200	1.03008700	-0.71483700
N	1.28813600	1.15040500	-1.33767400
H	1.76272600	4.23975100	-0.06018200
H	3.74172700	2.52484500	0.40956100
C	-0.67273300	2.40800000	-0.75869800
C	-1.71647800	3.26963900	-0.31633700
N	-1.15832000	1.16355200	-0.87192400
C	-2.84275000	2.46445200	-0.13975600

H	-1.64216500	4.33696100	-0.16109800
C	-2.47476400	1.11671700	-0.47191100
H	-3.81793600	2.77578900	0.21076000
C	2.53260400	-1.45552700	-0.70346000
C	2.92626100	-2.78907300	-0.28891100
C	1.79228200	-3.58562100	-0.30837100
C	0.70775300	-2.75947100	-0.75366600
N	1.21943400	-1.53304000	-1.06534000
H	3.92183700	-3.07316700	0.02510300
H	1.72403200	-4.62427400	-0.01234600
C	-0.73787300	-2.71970200	-0.65831100
C	-1.82887700	-3.53057600	-0.19842600
N	-1.21056300	-1.46457300	-0.85914400
C	-2.93508400	-2.70038000	-0.09679100
H	-1.78537900	-4.58525500	0.03955000
C	-2.52252100	-1.37071700	-0.47687900
H	-3.92644400	-2.97009000	0.24237500
C	3.16843200	-0.20456400	-0.51562300
C	-3.15782100	-0.11635200	-0.30568800
Ni	0.02045900	-0.16929300	-1.19051700
C	4.54438200	-0.15306300	0.06445600
C	5.64877000	0.07943700	-0.78014400
C	4.73654700	-0.31223900	1.45279700
C	6.93106100	0.13238700	-0.22566300
C	6.03549200	-0.25052700	1.96819500
C	7.14584600	-0.03585900	1.14586100
H	7.78265400	0.31019300	-0.87971000
H	6.18316400	-0.36760800	3.04014700
C	-4.57192100	-0.08842200	0.17551400
C	-4.84206500	-0.08224100	1.55788600
C	-5.62291000	-0.05960100	-0.76104100
C	-6.17433400	-0.05040000	1.98159300
C	-6.94011900	-0.02796100	-0.29377500

C	-7.23673700	-0.02609800	1.07278400
H	-6.38663100	-0.04241000	3.04893000
H	-7.75417200	-0.00214700	-1.01558400
C	-3.71275800	-0.10613400	2.56264900
H	-3.09774500	-1.00630000	2.44469900
H	-3.04507200	0.75292400	2.42726200
H	-4.09444300	-0.08490500	3.58790400
C	-5.32781700	-0.05565100	-2.24354300
H	-4.72778300	0.81824500	-2.52457300
H	-4.75037900	-0.94116200	-2.53504300
H	-6.25008400	-0.04004900	-2.83217400
C	-8.66905500	-0.02611900	1.55448400
H	-9.04955600	-1.05088800	1.66130200
H	-8.76417500	0.45929800	2.53186200
H	-9.32917300	0.49355100	0.85147100
C	5.44626900	0.27510900	-2.26583400
H	4.89516100	-0.56388500	-2.70640900
H	4.85819300	1.17837100	-2.47285800
H	6.40329800	0.37002200	-2.78828200
C	3.55766100	-0.52492600	2.37594300
H	2.80164000	0.25848900	2.24334600
H	3.05704200	-1.47838400	2.17132400
H	3.87159900	-0.52225700	3.42436300
C	8.54314100	-0.01001300	1.72063100
H	8.98855400	-1.01398200	1.72007700
H	9.20568900	0.63908900	1.13768600
H	8.54494400	0.34526900	2.75689000
C	0.77943500	3.20954500	-2.69950200
C	2.18363500	3.16735700	-3.34213500
H	2.92761400	3.68794400	-2.73566100
H	2.49070500	2.12462900	-3.46003500
H	2.15214100	3.63701600	-4.32976600
C	-0.23331500	2.50869500	-3.63243900

H	0.01536500	1.44541700	-3.69370200
H	-1.25726700	2.60415900	-3.26581500
H	-0.17786900	2.94779500	-4.63332300
C	0.37823800	4.61554000	-2.51260500
N	0.07129300	5.72141200	-2.33472600

2a

C	0.69601100	2.73481100	-0.78956600
C	1.67288600	3.40389400	0.15449700
C	2.65303600	2.53697900	0.46566500
C	2.33329900	1.23008700	-0.13964100
N	1.24174700	1.36830200	-0.90985100
H	1.59538300	4.44367200	0.44893900
H	3.51151900	2.71306200	1.10159800
C	-0.73583200	2.56429500	-0.28823900
C	-1.77476400	3.40458400	0.20685700
N	-1.18711500	1.30995400	-0.36991200
C	-2.86775300	2.57588100	0.44963000
H	-1.71403600	4.47414400	0.35230800
C	-2.48361500	1.23911000	0.10551400
H	-3.82851600	2.86171300	0.85692600
C	2.33199200	-1.23318400	-0.14541200
C	2.65035000	-2.54364100	0.45287700
C	1.66918900	-3.40781800	0.13735000
C	0.69299300	-2.73273500	-0.80313500
N	1.24026200	-1.36628400	-0.91634500
H	3.50872800	-2.72402000	1.08774000
H	1.59060800	-4.44904800	0.42632400
C	-0.73865000	-2.56319800	-0.30089900
C	-1.77867300	-3.40489200	0.18950600
N	-1.18848100	-1.30793600	-0.37607000
C	-2.87081100	-2.57623700	0.43622300
H	-1.71921600	-4.47527700	0.32929600
C	-2.48506800	-1.23810900	0.09928400

H	-3.83205600	-2.86314100	0.84159900
C	2.94779900	-0.00269400	0.19134800
C	-3.12630800	0.00033700	0.31765000
Ni	-0.00981800	0.00149100	-0.83308300
C	4.14626400	-0.00483200	1.08859900
C	5.44323900	0.00952600	0.53716800
C	3.98000600	-0.01434000	2.48989100
C	6.55095200	0.01199700	1.39110600
C	5.11159600	-0.01172300	3.31225800
C	6.40565800	-0.00184800	2.78189500
H	7.55097200	0.02630500	0.96151300
H	4.98041300	-0.01575800	4.39279300
C	-4.51244700	-0.00027200	0.87505500
C	-4.70341600	-0.00531400	2.27018800
C	-5.61291700	0.00976800	-0.00262800
C	-6.01031800	-0.00151900	2.76715900
C	-6.90216800	0.01317500	0.53789100
C	-7.12197500	0.00456400	1.91884500
H	-6.16285800	-0.00199800	3.84462000
H	-7.75549800	0.02408600	-0.13729800
C	-3.51900400	-0.00998800	3.20964100
H	-2.88724200	-0.89084100	3.04435400
H	-2.88422600	0.86974000	3.04988700
H	-3.84270000	-0.01276200	4.25474200
C	-5.40106800	0.02101800	-1.49925800
H	-4.82992500	0.90368200	-1.81125100
H	-4.82977000	-0.85690800	-1.82408400
H	-6.35488800	0.02479500	-2.03522300
C	-8.52471100	-0.02640300	2.47981000
H	-8.87351500	-1.05935000	2.61231500
H	-8.57660000	0.46218400	3.45883600
H	-9.23524800	0.47335000	1.81252200
C	5.63217500	0.02707600	-0.96281500

H	5.16467400	-0.84630800	-1.43346600
H	5.16182800	0.90939100	-1.41373500
H	6.69353500	0.03205400	-1.23060900
C	2.59409600	-0.02193700	3.09544700
H	2.01428900	0.85367200	2.77926300
H	2.02278900	-0.90222300	2.77676900
H	2.63932600	-0.02354300	4.18904800
C	7.61493900	-0.03403000	3.68774700
H	7.91903100	-1.06700900	3.90436600
H	8.47412700	0.46757600	3.22916600
H	7.41071700	0.45197800	4.64806100
C	0.69147000	3.44929500	-2.22089900
C	2.10961100	3.47494200	-2.83179800
H	2.81830500	4.00656400	-2.19262700
H	2.45640300	2.44618000	-2.96102000
H	2.08698300	3.96695500	-3.80881800
C	-0.27809800	2.73156700	-3.18569200
H	0.02334100	1.68398200	-3.27647400
H	-1.30791300	2.76552600	-2.82326800
H	-0.23822500	3.20204200	-4.17293200
C	0.23207800	4.83398100	-2.00892900
N	-0.12103600	5.92205900	-1.80843100
C	0.68753600	-3.43983600	-2.23813600
C	2.10561100	-3.46407500	-2.84924500
C	-0.28115700	-2.71597500	-3.19921900
H	2.81368500	-3.99988800	-2.21290200
H	2.08230000	-3.95096400	-3.82881300
H	2.45366600	-2.43508300	-2.97312200
H	-1.31096900	-2.75020300	-2.83680600
H	0.02181800	-1.66840400	-3.28492400
H	-0.24211500	-3.18165000	-4.18876600
C	0.22650200	-4.82504600	-2.03327300
N	-0.12784000	-5.91373300	-1.83831300

Selected absorption wavelengths, energies, and oscillator strengths (f) of 2a calculated at the M06/6-31G(d)+SDD//B3LYP-D3/6-31G(d)+SDD level of theory.

Excited State 1: Singlet-A 1.8500 eV 670.17 nm f=0.0026 <S**2>=0.000

169 ->185 0.11936

169 ->188 -0.19458

173 ->188 0.13363

178 ->185 0.15996

178 ->188 -0.22009

183 ->185 0.46660

183 ->188 -0.34879

This state for optimization and/or second-order correction.

Total Energy, E(TD-HF/TD-DFT) = -2199.89793617

Copying the excited state density for this state as the 1-particle RhoCI density.

Excited State 2: Singlet-A 2.1748 eV 570.09 nm f=0.0042 <S**2>=0.000

170 ->185 -0.12632

170 ->188 0.21684

174 ->188 0.13791

181 ->185 -0.38318

181 ->188 0.45789

183 ->184 -0.17508

Excited State 3: Singlet-A 2.1957 eV 564.67 nm f=0.0000 <S**2>=0.000

175 ->185 -0.40637

175 ->188 0.52789

178 ->185 -0.12213

178 ->188 0.16151

175 <-188 -0.10937

Excited State 4: Singlet-A 2.2784 eV 544.17 nm f=0.0054 <S**2>=0.000

181 ->188 0.12892

183 ->184 0.68084

Excited State 5:	Singlet-A	2.5380 eV	488.51 nm	f=0.0001	<S**2>=0.000
171 ->185	0.16419				
171 ->188	-0.25654				
172 ->185	0.14928				
172 ->188	-0.23511				
173 ->185	0.22274				
173 ->188	-0.33981				
178 ->185	0.23145				
178 ->188	-0.26364				
182 ->184	0.10040				
183 ->185	-0.11918				

Excited State 6:	Singlet-A	2.7064 eV	458.11 nm	f=0.0360	<S**2>=0.000
173 ->185	-0.12028				
173 ->188	0.15877				
181 ->184	0.13044				
182 ->184	0.51325				
183 ->185	-0.29729				
183 ->188	-0.21415				

7. References

1. Ito, T.; Hayashi, Y.; Shimizu, S.; Shin, J.-Y.; Kobayashi, N.; Shinokubo, H. *Angew. Chem. Int. Ed.* **2012**, *51*, 8542–8545.
